# High prevalent human papillomavirus infections of the oral cavity of asymptomatic HIV-positive men

**DOI:** 10.1186/s12879-019-4677-9

**Published:** 2020-01-10

**Authors:** Rocío Méndez-Martínez, Silvia Maldonado-Frías, Salvador Vázquez-Vega, Yanink Caro-Vega, José Guadalupe Rendón-Maldonado, Miriam Guido-Jiménez, Brenda Crabtree-Ramírez, Juan G. Sierra-Madero, Alejandro García-Carrancá

**Affiliations:** 1 División de Investigación Básica, Instituto Nacional de Cancerología, Av. San Fernando #22, Tlalpan, 2do piso, Torre de Investigación Básica, 14080 CDMX México City, Mexico; 2 Doctorado en Biotecnología & Doctorado en Ciencias Biomédicas, Facultad de Ciencias Químico-Biológicas, Universidad Autónoma de Sinaloa, Culiacán, Sinaloa Mexico; 3 División de Estudios de Posgrado e Investigación, Facultad de Odontología, Universidad Nacional Autónoma de México, Universidad 3000, Coyoacán, Copilco Universidad, 04360 Ciudad de México, CDMX, México City, Mexico; 4 Unidad de Investigación Epidemiológica y en Servicios de Salud, Centro Médico Nacional Siglo XXI, Instituto Mexicano del Seguro Social, Av. Doctores 330, Cuauhtemoc, 06720 Ciudad de México, CDMX Mexico; 5 Instituto Nacional de Ciencias Médicas y Nutrición “Salvador Zubirán” (INCMNSZ), Secretaría de Salud, México City, Mexico; 6 Facultad de Ciencias Químico-Biológicas- UAS, Universidad Autónoma de Sinaloa, Calz. de las Américas Norte 2771, Burócrata, 80030 Culiacán, Sinaloa Mexico; 7 Unidad de Investigación Biomédica en Cáncer, Instituto de Investigaciones Biomédicas, Universidad Nacional Autónoma de México & Instituto Nacional de Cancerología, Av. San Fernando #22, Tlalpan, 2do piso, Torre de Investigación Básica, 14080 CDMX México City, Mexico

**Keywords:** HIV +, HPV, Types and variants MSM, Oral cavity, Anal canal

## Abstract

**Background:**

Incidence of anal and oral infections with Human Papillomavirus (HPV) is increasing, particularly among Human Immunodeficiency Virus-positive (HIV+) men. HPV type 16 has exhibited the highest incidence and only limited data is available on other prevalent types, variants of HPV16, as well as associated factors. We were interested in identifying prevalent HPV types, variants of type 16, as well as factors associated with HPV16 infections in the oral cavity of HIV+ men who have sex with men (MSM).

**Methods:**

A cross-sectional study of oral cavity samples from HIV+ MSM, that in a previous study were identified as positive for HPV16 in the anal canal. Cells from the oral cavity (102 samples, paired with 102 from the anal canal of same patient) were used to extract DNA and detect HPV infections using INNO-LiPA HPV Genotyping Extra II, and PCR. From these, 80 samples (paired, 40 anal and 40 oral) were used to identify variants of type 16 by sequencing. Statistical differences were estimated by the X^2^ test, and *p* values equal to or less than 0.05 were considered significant. SPSS ver. Twenty-four statistical software (IBM Corp) was used.

**Results:**

We found a high prevalence of High-Risk HPV (HR-HPV) and Low-Risk HPV (LR-HPV). Patients were positive in the oral cavity for HR types; 16, 39 and 18 (80.4, 61.8 and 52.9% respectively) and LR types 11 and 6 (53.9 and 34.3% respectively). Surprisingly, only European variants of type 16 were found in the oral cavity, although American Asian (22.5%) and African (2.5%) variants were identified in the anal canal. The analysis showed that CD4 counts could be the most important risk factor associated with HR-HPV infections in the oral cavity, anal canal or both anatomical regions. The risk of infection of the oral cavity with type 18 increased in men diagnosed with HIV for more than 6 years.

**Conclusions:**

Prevalence of both HR and LR HPV’s in the oral cavity of Mexican HIV+ MSM is very high. The fact that only European variants of HPV16 were found in the oral cavity suggest a possible tropism not previously described.

## Background

HPV’s are recognized as one of the major causes of sexually transmitted infections related to cancer worldwide, and have been detected in cervical, anal and oral high-grade intraepithelial lesions [1–3]. Among them, HPV type 16 is the most common one, causing the majority of cervical and anogenital cancer as well as an important proportion of head and neck cancers [4–10]. Oral infection with HPV16, is associated with oropharyngeal cancer (OPC) and the oral sex play an important role in their acquisition [11, 12].

Infection with human immunodeficiency virus (HIV) affects 36.9 million people worldwide with more than 1.7 million persons in Latin America [13, 14]. HIV infections cause a marked decrease in host defenses, generating conditions for an increased rate of coinfections [15]. HIV+ patients have a high risk of developing cancer due to coinfections by HR-HPV [8, 16]. In particular, the presence of HPV16 is relevant since it is the major type associated to cancer in HIV+ patients [17–20]. The persistent oral infection of these patients have with HR-HPV can lead to development of head and neck cancer (HNC), with a minor association with oral cancer and a major role with OPC [21–23].

The prevalence of oral HR-HPV infection is relatively low among the general population, accounting for 3.5%, [24] while a higher prevalence of up to 50% has been reported among HIV + MSM, showing high vulnerability by coinfection in different anatomical regions [8, 21, 25]. Persistence of infections and progression to High grade Squamous Intraepithelial Lesion (HSIL) and cancer development have been are associated with variants of HPV16 [26–30].

Variants of HPV types reflect an evolutionary history of viral genomes associated with the rapid expansion of the human populations they infect and that can be used for epidemiological and etiologic studies to investigate to transmission of this virus within and among populations, as well as their presence on different anatomical regions. HPV16 variants have been grouped on five main groups: European (E), Asian (As), Asian American (AA), African-1 (Af1), African-2 (Af2), that show a particular geographic distribution, often clustering at specific parts of the world, such as Africa East Asia, North and Latin-America [31, 32]. Several studies have observed a high risk of cervical cancer associated with non-European variants of HPV-16. Infection with non-European variants increases the risk from 2 to 9 times for the development of cervical cancer [33, 34]. Studies conducted in Brazil [35] and the United States [29], are found that non-European variants are associated with persistent infection and that these variants are most frequent in HSIL [29, 36]. On the other hand, Berumen et al. in 2001 found that the AA variants confer a higher risk for development of cervical cancer compared to variants European, and that about a quarter of cervical cancer cases in Mexico are attributed to these variants [33]. Similarly, Xi et al. in 2007, showed that women infected with the variants Af2 and AA have 2.7 (95% CI 1.0–7.0) and 3.1 (95% CI, 1.6–6.0) times higher risk of IAS 3 compared to Women infected with European variants [37].

Previously our group determined that HIV+ MSM exhibited a multiplicity of associated viral types, in addition to HPV16 in anal canal and among HPV16-positive patients, European variants were the most prevalent [38]. We now aimed to determine the prevalence of HR and LR types of HPV in addition to type 16 and determine the nature of HPV16 variants, as well as clinical factors associated with HPV infections in the oral cavity of HIV+ MSM that are positive for HPV16 in the anal canal of same patients. In addition, the information on HPV prevalence and genotypes in Mexican HIV+ patients are necessary for HPV related anal and oral neoplasia prevention.

## Methods

### Samples

Three hundred twenty-four patients were recruited in the HIV Clinic at Instituto Nacional de Ciencias Médicas y Nutrición “Salvador Zubirán” located in Mexico City. Selected samples have the characteristic of being positive for type 16, determined in a previous study of the prevalence of HPV in the anal canal of HIV+ MSM [38].

In this cross-sectional study, we analysed 204 samples (102 HPV16+ in the anal canal and 102 paired from the oral cavity of the same patients) to detect simultaneous co-infections of other HPV types. From these, only 80 with an enough sample quantity, (paired 40 anal and 40 oral) were obtained and used to identify variants of type 16. Samples were obtained and processed as previously described by Mendez et al., 2014 [38].

### Data collection

Socio-demographic, clinical and sexual behaviour information was collected using a self-applied written questionnaire. Clinical information was verified and completed with information available in the clinic records. The age of participants, education, employment, time since HIV diagnosis, time with anti-retroviral therapy treatment (time with ART), regimen of ART initiation (regimen of anti-retroviral initiation), were asked. CD4 (cells/mm^3^) levels, and viral suppression (defined as HIV-RNA <200 copies/ml), were evaluated.

### Typing HPV

Extraction and purification of DNA was performed with the Genomics Wizard kit (PROMEGA; Madison, WI 53711–5399 USA). For identification of viral types present in the samples, we used the INNO-LiPA® HPV Genotyping Extra II kit (INNOGENETICS Heiden Germany), which detects 32 different HPVs. HR-HPV (16, 18, 31, 33, 35, 39, 45, 51, 52, 56, 58, 59, 66 and 68) and LR-HPV (6, 11, 40, 42, 43, 44, 54, 61, 62, 67, 81, 83 and 89) and the additional types; 26, 53, 70, 73 and 82.

### Identification of HPV-16 variants

The HPV16+ samples were subjected to a polymerase chain reaction (PCR) to identify variants within E6 and the long control region (LCR) with specific primers for HPV16 (5′ GCAACAGTTACTGCGACGTG3´/5’GGACACAGTGGCTTTTGACA 3′; product size: 215 pb for E6 and 5’TCAACCGAATTCGGTTGCAT3´/ 5’ACCTTTACACAGTTCATGTA3´; product size: 363 pb). The PCR products were sequenced using the Big Dye terminator kit and equipment Applied Biosystems 3500/3500xL Genetic Analyzer. Analysis of the sequences was done with the Chromas Program and the alignment of them program Multiple Sequence Alignment-Clustalw.

### Statistical analyses

A descriptive statistical analysis was performed to summarize the sociodemographic characteristics of the selected population. The quantitative variables with normal distribution, were expressed as mean and standard deviations (SD). For data with free distribution, the median and Interquartile Ranges (IQR) were used. Qualitative variables were categorized and expressed by frequency and percentage. The prevalence of HPV was reported as the proportion of anal, oral and anal-oral samples positive for each identified viral type. (the positivity of the high risk and low risk HPV was considered when the proportion of positive cases of HPV in which the specific HPV was detected). The bivariate (Pearson’s X^2^ test) and multivariate analysis (bivariate logistic regression) of risk factors (age, education, employment, time since HIV diagnosis, time with Anti-Retroviral Treatment (ART), regimen of ART initiation, CD4 levels and viral suppression) were performed specifically for each type of HPV and as group: LR HPV (6, 11, 40, 44 and 70), and HR HPV (16, 18, 39, 51, 52, 66 and 68), HPV 16–18 and “Other HR HPV” (39, 51, 52, 66 and 68 that excluded the types 16 and 18). From the multivariate analysis adjusted for the risk factors, above mentioned, for infection by type of HPV (individual or in group), in the anatomical regions studied, those that had statistical significance for each logistic regression model were represented. For each variable, the alpha level used for the statistical analyses’ significance was *p* < 0.05. SPSS ver. Twenty-four statistical software (IBM Corp) was used.

## Results

### Sociodemographic characteristics of participants

One hundred two patients were included in the study. The age of participants ranged from 21 to 61 years, average was 38.4 ± 8.0 years. Median time since diagnosis of HIV was 4.3 years (range, 2.25–7.85 years), while median of ART was 2.16 years (range, 0.33–4.93 years); 59.8% of participants have an ART with a regimen based Non-Nucleotide Reverse Transcriptase Inhibition (NNRTIs). On average, the CD4 count was 428.76 ± 236.23, and nearly 80% of the participants reported viral suppression (Table 1).

**Table 1:** Sociodemographic characteristics from the 102 participants in this study

Variable	*N*	(%)	MCT	DM
Age (years))			38.43	± 8.0
≤ 35	37	36.3		
36–41	30	29.4		
≥ 42	35	34.3		
Education (years)			12	(11.63–12.00)
≤ 12	57	55.9		
≥ 13	45	44.1		
Employment				
No	31	30.4		
Yes	71	69.6		
Time since HIV diagnosis (years)			4.3	(2.25–7.85)
≤ 3.16	34	33.3		
3.17–6.17	33	32.4		
≥ 6.18	35	34.3		
Time with ART (years)			2.16	(0.33–4.93)
≤ 0.65	36	35.3		
0.66–4.0	31	30.4		
≥ 4.1	35	34.3		
Regimen of ART				
NNRTIs	61	59.8		
PI	33	32.4		
Other	8	7.8		
CD4 count			428.76	± 236.23
≤ 200	18	17.6		
201–499	53	52		
≥ 500	31	30.4		
Viral suppression				
No	21	20.6		
Yes	81	79.4		

Note: *MCT Measure of central tendency and *DM* Dispersion measure (Mean ± Deviation Standard Deviation [SD] or Median and Inter Quartile Range ([IQR] = P°25-P°75) were reported for continuous variables. N = Frequency. (%) = Percentage reported for categorical variables. Time with ART = Time with anti-retroviral therapy treatment. Regimen of ART initiation = Regimen of anti-retroviral initiation. NNRTIs: Non-Nucleoside Reverse Transcriptase Inhibition. PI: Protease inhibitor. Viral suppression defined as HIV-RNA <200 copies/mL. (Bavinton et al., 2018)

### Prevalence of LR & HR HPV in the oral cavity

We detected 5 LR types; 6, 11, 40, 44 and 70, and 7 HR types; 16, 18, 39, 51, 52, 66 and 68. HPV11 was the most prevalent LR type; 65.7% (67 patients) in the anal canal and 53.9% (55 patients) in the oral cavity, while 37.3% (38 patients) exhibited type 11 in both regions.

Regarding HR HPV, HPV16 was the most prevalent type of HR-HPV type in the oral cavity with 80.4% (82 patients). HPV39 was the second most prevalent HR type; 58.8% (60 patients) in the anal canal and 61.8% (63 patients) in the oral cavity, while 33.3% (34 patients) exhibited it in both regions.

HPV52 type was the third; 56.9% (58 patients) in the anal canal and 49% (50 patients) in the oral cavity, while 26.5% (27 patients) in both regions. The rest of HR-HPV types (18, 51, 66, and 68) were less prevalent in anal canal, oral cavity or both anatomical regions.

**Fig. 1:**
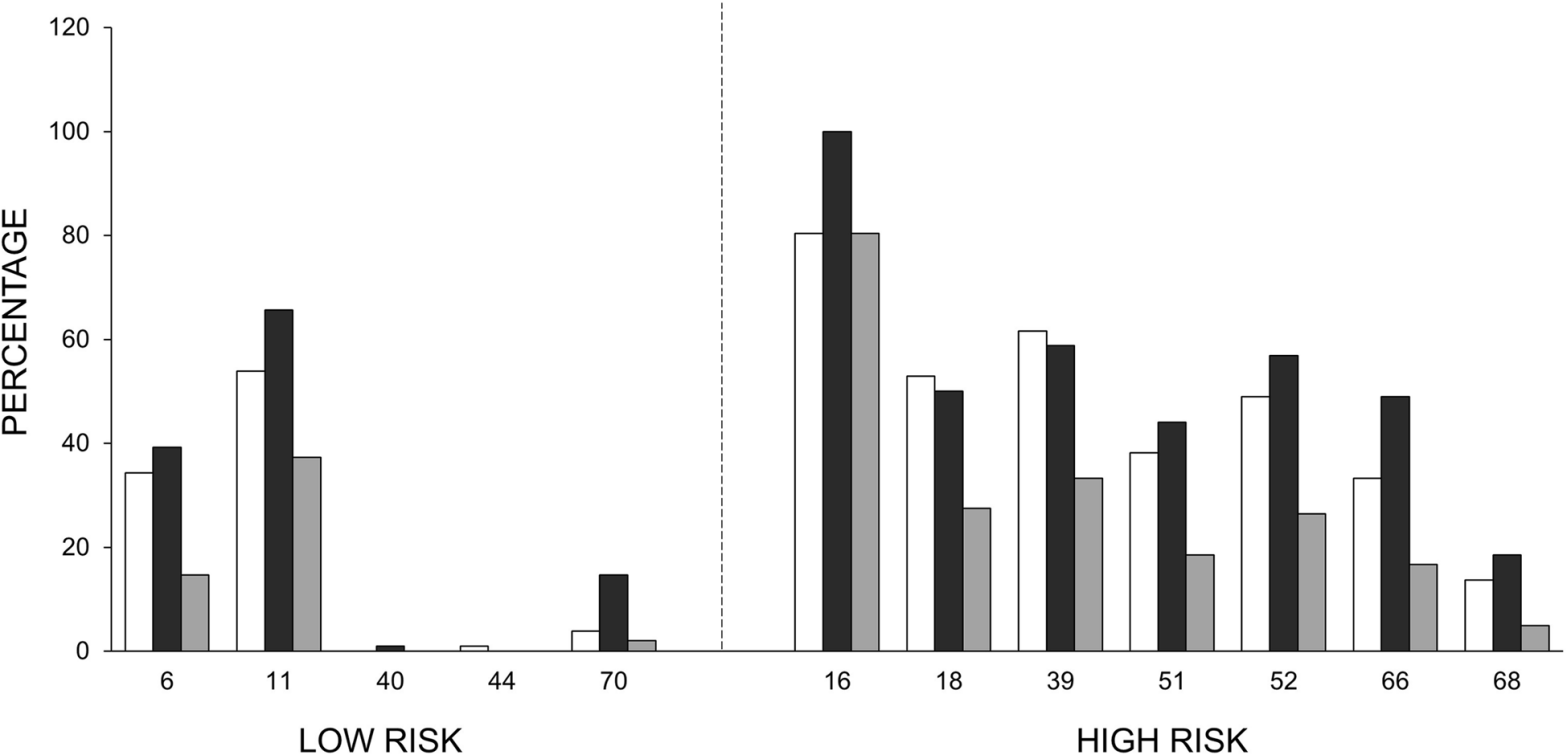
The prevalence of different low-risk or high-risk viral types is indicated according to their localization in two different anatomical regions; the anal canal (black bar), the oral cavity (white bar), or in both regions (grey bar)

Considering LR types, while 80.4% (82/102) of the anal samples were positive, only 66.7% (68/102) of the oral samples contained LR HPV. The positivity for LR in both anatomical regions was 55.9% (57/102) (Fig. 1).

HPV 16–18 were present almost equal in anal and oral cavity (45.1 and 50.0%, respectively) and in 21.6% in both anatomical sites. The group of “Other HR HPV” (HPVs 39, 51, 52, 66 and 68) showed high prevalence in the samples from the anal canal, oral cavity and simultaneously in both anatomical regions (92.2 and 80.4% and 72.5%, respectively) (Table 2).

**Table 2:** Prevalence of different HPV in the anal canal, the oral cavity, or both anatomical regions

Viral type	Anal canal		Oral cavity		Both	
	%	N	%	n	%	N
Low-risk HPV types						
6	39.2	40	34.3	35	14.7	15
11	65.7	67	53.9	55	37.3	38
40	1	1	0	0	0	0
44	0	0	1	1	0	0
70	14.7	15	3.9	4	2	2
LR-HPV	80.4	82	66.7	68	55.9	57
High-risk HPV types						
16	100.0	102.0	80.4	82	80.4	82
18	50.0	51	52.9	54	27.5	28
39	58.8	60	61.8	63	33.3	34
51	44.1	45	38.2	39	18.6	19
52	56.9	58	49.0	50	26.5	27
66	49.0	50	33.3	34	16.7	17
68	18.6	19	13.7	14	4.9	5
HPV16–18	45.1	46	50.0	51	21.6	22
Other HR HPV	92.2	94	80.4	82	72.5	74

LR HPV = 6, 11, 40, 44 and 70 types. HPV 16–18 = includes both HPV types

Other HR HPV = 39, 51, 52, 66 and 68, and excluding types 16 and 18

### Risk factors associated with HPV infections in the anal canal, the oral cavity, and both sites

To evaluate the possible association of several risk factors with or without probability of HPV infection, we performed a logistic regression adjusted by age of participants, education, employment, time since HIV diagnosis, time with anti-retroviral therapy treatment (time with ART), regimen of ART initiation (regimen of anti-retroviral initiation), CD4 (cells/mm^3^) levels, and viral suppression (defined as HIV-RNA <200 copies/ml). Only those risk factors that had statistical significance for each logistic regression model are shown in Table 3.

**Table 3:**
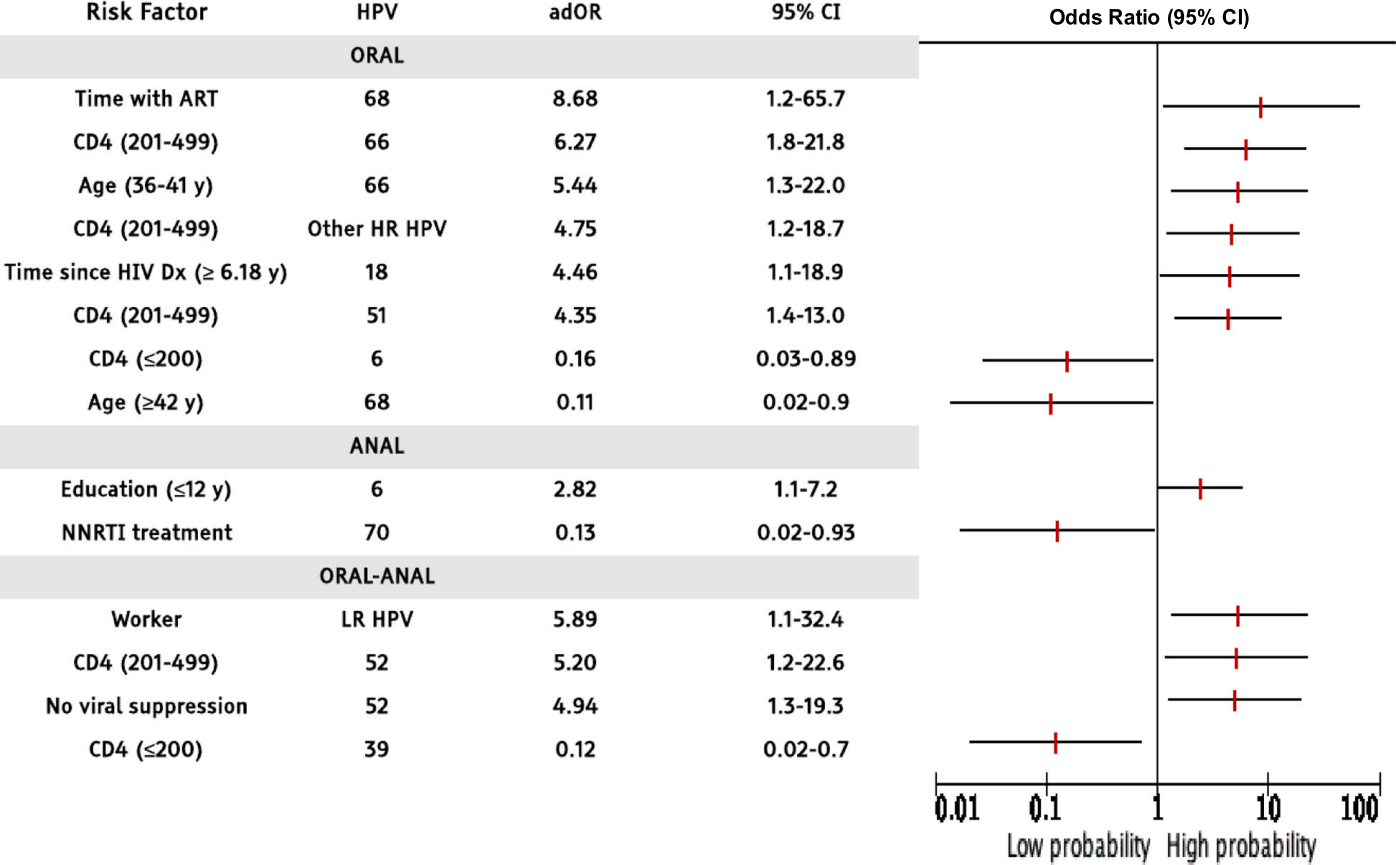
Risk factors associated with low- and high-risk HPV in a Mexican MSM HIV+ population

Note: Odds Ratios were adjusted (adOR) for all the variables included in this study [age of participants, education, employment, time since HIV diagnosis, time with anti-retroviral therapy treatment (time with ART), regimen of ART initiation (regimen of anti-retroviral initiation), CD4 (cells/mm^3^) levels, and viral suppression (defined as HIV-RNA < 200 copies/ml)]. Only those risk factors that had statistical significance (*p* < 0.05) for each logistic regression model are presented in the table (even confidence intervals were ample in some instances, they were included if significant). 95% Confidence Intervals (95% CI)

Concerning HR HPV types, in oral infection with HPV 68 type was associated with time under ART treatment (6 months and a half to 4 years), while HPV 68 infection in the same anatomical region was less associated in patients older than 42 years.

In general, the risk of infection of the oral cavity with 66 and 51 types increases with CD4 counts between 201 and 499. Whereas oral infection with 66 type correlates with age (36–41 years). We found that the risk of infection of the oral cavity with type 18 increased in men diagnosed with HIV for more than 6 years. HPV 52 infection at both anatomical regions in men with CD4 (201–499) and without viral suppression was associated. The “Other HR types” infection risk was high associated in patients with CD4 counts between 201 and 499. On the contrary, HPV 39 infection in both anatomical sites (anal & oral) in patients with CD4 < 200 was low associated. Regarding HPV 6 infection was related in the anal region among men with lower education (fewer than 12 years), while oral infection with this same viral type was low linked in patients with a CD4 count < 200. The HPV 70 infection in the anal region was less probable in patients under NNRTIs treatment regimen. The LR-HPV infection (as a group) were correlated in the oral-anal region of men who had work (Table 3).

### Identification of variants of HPV16

**Fig. 2:**
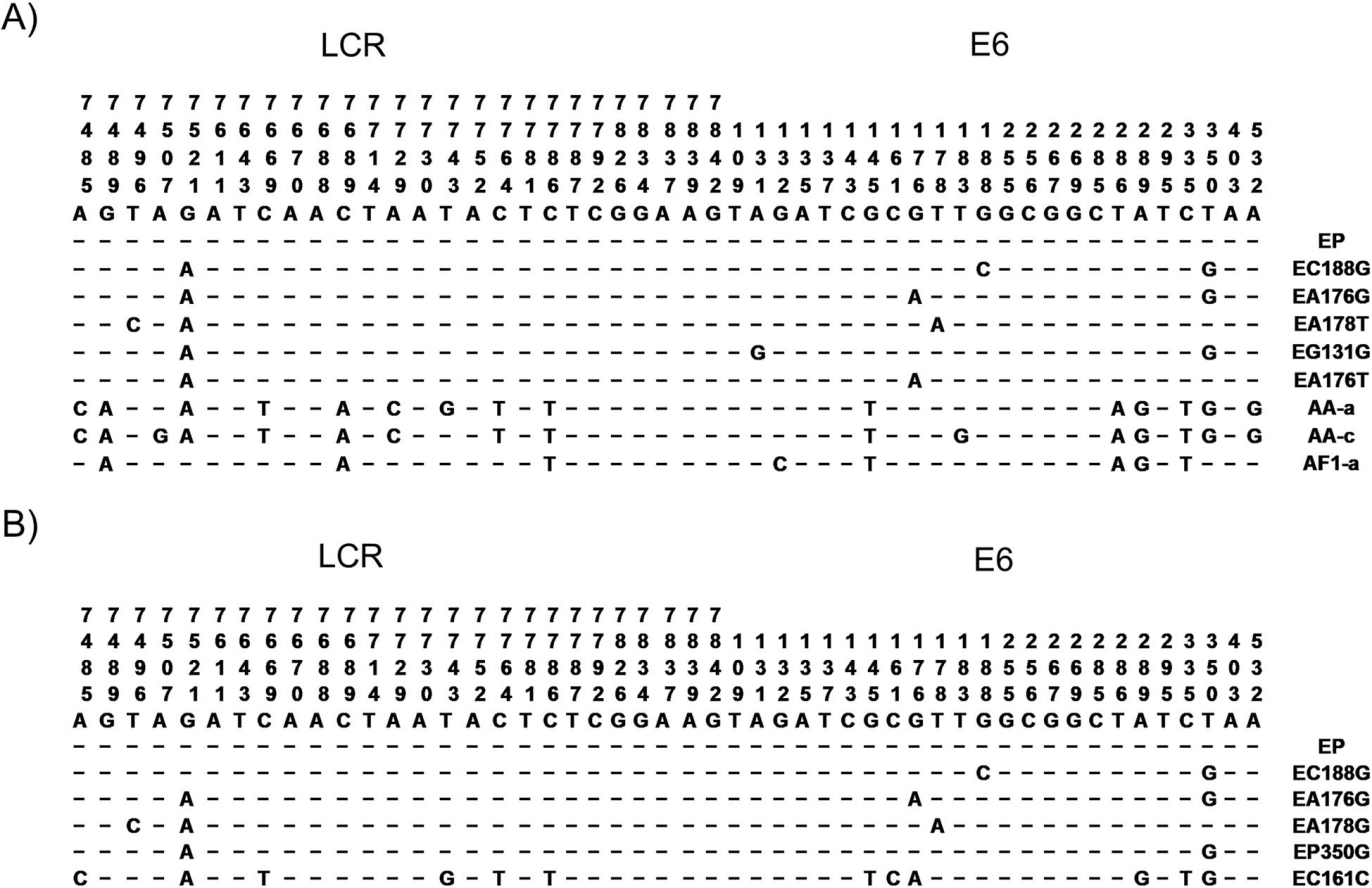
Representative HPV-16 variant sequences along the Long Control Region (LCR) and E6 gene. Summary of variant sequences identified in the anal canal (**a**) and the oral cavity (**b**) of HIV+ MSM. European prototype (EP), Asian-American (AA) and African (Af1) variants were identified and classified as previously described (Yamada et al., 1996)

From 204 samples (102 HPV16+ in the anal canal and 102 paired from the oral cavity of same patients), only 80 with enough sample quantity, (40 anal and 40 paired oral) were obtained and used to identify variants of type 16. Sequence analysis of PCR products from the E6 and LCR regions of type 16-positive samples showed three types of variants (European; E, Asian-American; AA, and African; Af) were present in the anal samples. The most frequent variant was European (75%/30 patients), followed by Asian- American (22.5%/9 patients), and African (2.5%/1 patient). Surprisingly, in the oral cavity only European variants were detected (Fig. 2). The analysis of European variants vs Asian-American variants, and African variants, indicated this is not statistically significant (data not shown).

**Fig. 3:**
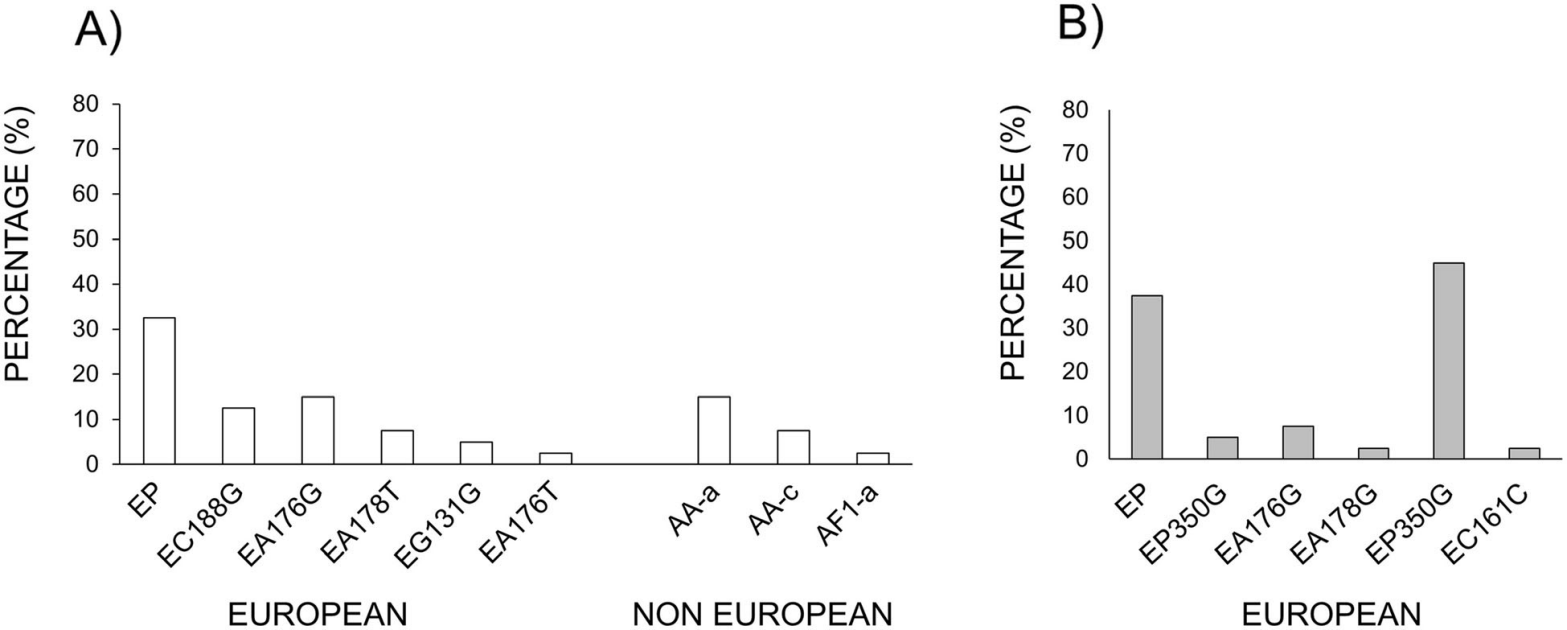
Summary of variants of HPV type 16 in the anal canal and the oral cavity. **a** anal canal, (**b**) oral cavity. The statistical analysis of European variants vs Asian-American variants, and African variants showed no significant differences

In the anal canal, the European Prototype (EP) was the most prevalent variant of HPV16 (13 patients), followed by EA176G (six patients), EC188G (five patients), EA178T (three patients), and EG131G (two patients). Regarding non-European variants, the American-Asian AA-a (six patients) and AA-c (three patients) and the African Af1-a (one patient) were the most prevalent ones. In the oral cavity, the most prevalent variant of type 16 was the European 350G (18 patients), followed by the European Prototype (EP) (15 patients), EA176G (three patients), and EA178G and EC161G (Fig. 3a and b). Only 20% (eight patients) had the same variant in both regions: five patients presented the European-variant Prototype in both regions, two patients presented the EC188G variant in both regions, and one patient presented the EA176G variant in both anatomical regions.

## Discussion

HPV plays a decisive role in the development of anal, cervical squamous cell and oropharyngeal cancer; the most prevalent HPV types associated with these cancer and precancerous lesions is HPV16. Although several studies show multiple infection of HPV present in the anal canal [8, 20, 27, 38–41], the relationship of theses viral types in the oral cavity is unclear, reports suggest that oral HPV infection in MSM, differs from anogenital HPV infection [39, 40]**.** This study investigated for the first time the multiple infection of HPV in the anal canal and the oral cavity of HPV16+ and HIV+ MSM of same patients.

The mechanisms of anal HPV infection are possibly due to both, sexual and not sexual transmission. The sexual transmission shown specific risk factors for anal HPV infection including younger age patients HIV+, and not sexual transmission includes insertion of transiently infected fingers or toys, as well as shedding from other infected genital sites [42–45]. On the other hands, recently is suggested that HPV infections in the oral cavity also are associated with sexual practices, however evidences show that vertical transmission is present in newborns from HPV + mothers, indicating that HPV can be acquired at an early age and may remain latent for years, and has been report that exists horizontal transmission, which can mediated by saliva [46]. It has been show that, the infection by HPV in oral cavity has higher prevalence by 16 type in young healthy and HIV+ men [47–49]**.**

This cross-sectional study, from HIV+ Mexican MSM patients, showed that around two-thirds of these patients had both anal and oral infection with oncogenic HPV types We observed a high prevalence of HPV coinfections in the anal canal, confirming our previous report [38]. We found in the anal canal, that type 16 was the most prevalent, followed by type 39. Type 16 predominated in the oral cavity too. We found a similar prevalence of co-infections with LR and HR types of HPV in our study, as compared to previously published work [50, 52]. Prevalence of types 16/18 was similar to what has been shown in previous studies of patients in Europe and Western countries among HIV+ men [53]. Compared with heterosexual men (HM), the prevalence of HPV infection was much higher [54, 55].

Risk factors analysis showed that CD4 (201–499) count could be the most important risk factor associated with infection of oncogenic HPV types, in the oral cavity, anal region or both anatomical regions, (e.g. HPV-66, 52 and 51), we found that anal and oral infection with oncogenic HPV types were significantly associated with decreased CD4 counts and short HAART durations. HPV infection can cause significant pathology in all men, but MSM are at particularly susceptible.

On other hand it has been widely reported that variants of viral types determined among human populations correlate with geographic regions, and associated with different pathologic risks for the development of cancerous lesions [29, 33, 35, 37]. Although we observed a high prevalence of European variants of type 16 in the anal canal, we found only European variant in the oral cavity. It is important to note that tropism for these different anatomical regions is suggested. The involvement of HPV16 variants in the determination of the persistence and progression of viral infection can be explained by the fact that nucleotide variations in the HPV coding region could result in a decrease in the immunogenicity of viral proteins, favoring the persistence of infection in immunocompetent individuals. In fact, the evaluation of the integration of HPV16 in the host genome seems to be a good biomarker for predicting anal precancerous lesions in HIV positive men [34]. The presence of the EP350G variant in both anatomical regions could suggest an infection by the same virus. It is worth nothing that this variant is related to persistence, fundamental for the development of HPV-related cancer. It would be very important to follow-up in this high-risk population as well as to implement a program for their vaccination.

## Conclusions

In this study we found a high prevalence of HR and LR types of HPV, in addition to type 16 in the anal canal and the oral cavity of HIV+ MSM. Importantly, the fact that non-European variants of HPV16 were only found in the oral cavity suggests a tropism for different anatomical regions. In addition, the information on HPV prevalence and genotypes in asymptomatic Mexican HIV+ patients are necessary for HPV related anal and oral neoplasia prevention.

This finding supporting the importance of early prophylactic vaccination in this population to achieve maximum benefit. It is important to mention that the use of quadrivalent HPV (types 6, 11, 16, 18) vaccine in HPV + MSM prevented 95% of persistence anal HPV infections in young HIV-uninfected MSM [56]. Because the HPV vaccine appears to be safe in HIV+ men, a significant proportion of HIV + MSM in Mexico may potentially benefit from the vaccine, which might prevent HPV-associated anal or oral cancer.

Our data also support the need for establishing anal and oral screening systems for early detection of cancer among these patients and regular follow-up strategies, especially in HIV+ patients with high-risk HPV. Priority should be given to the identification and treatment of anal or oral lesions in MSM with HIV/AIDS.

## Data Availability

The datasets used and/or analyzed during the current study are available from the corresponding author on request. The sequencing data generated in this study has been deposited in the NCBI SRA database Accession: PRJNA591936; ID: 591936) (https://www.ncbi.nlm.nih.gov/bioproject/PRJNA591936).

## Abbreviations

ART: Anti-Retroviral Therapy
CI: Confidence Intervals
DNA: Deoxyribonucleic acid
HAART: Highly Active Antiretroviral therapy
HIV +: Human Immunodeficiency Virus-positive
HM: Heterosexual men
HNC: Head and Neck Cancer
HPV: Human Papilloma Viruses
HR: High Risk
HSIL: High grade Squamous Intraepithelial Lesion
IQR: Interquartile Ranges
LCR: Long Control Region
LR: Low Risk
MSM: Men who have Sex with Men
NNRTIs: Non-Nucleotide Reverse Transcriptase Inhibition
OPC: Oropharyngeal cancer
OR: Odds Ratios
PCR: Polymerase chain reaction
PI: Protease Inhibitor
SD: Standard deviations
